# Cardiac Conduction Defects in Systemic Lupus Erythematosus

**DOI:** 10.7759/cureus.10882

**Published:** 2020-10-10

**Authors:** Shayan Butt, Simra Kiran, Nida Qadir, Divya Menghani, Hammad Tanzeem

**Affiliations:** 1 Internal Medicine, Baptist Memorial Hospital, Oxford, USA; 2 Pulmonology and Critical Care, University of Cincinnati Medical Center, Cincinnati, USA; 3 Infectious Diseases, University of Louisville, Louisville, USA; 4 Medicine, Dow Medical College/Civil Hospital, Karachi, PAK

**Keywords:** cardiac lupus, anti-ro/ssa, anti-la/ssb

## Abstract

Systemic autoimmune conditions may cause morbidity and mortality. Systemic lupus erythematosus (SLE) is a prominent example of such diseases. It can result in conduction abnormalities due to accelerated atherosclerosis, vasculitis, or autoantibodies-induced myocarditis. Cardiac conduction abnormalities may produce sinus tachycardia, sinus bradycardia, prolonged QT intervals, atrial fibrillation, or atrioventricular (AV) nodal blocks. Neonatal lupus is sometimes associated with anti-Ro/SSA and anti-La/SSB antibodies, but their role remains a matter of controversy in adults.

## Introduction and background

Among all the cardiac manifestations of systemic lupus erythematosus (SLE), the main ones are coronary artery disease, myocardial dysfunction, pericardial effusion, and mitral regurge [1-4]. Cardiac involvement is observed in 50% of the cases [5]. These abnormalities are not very well understood and, most often, are asymptomatic. Anti-Ro/SSA antibodies have relevance to neonatal lupus [6], but their role is undefined in adults. In most cases, the patients are asymptomatic at presentation, and electrocardiographic abnormalities are linked to an autoimmune condition. Sinus tachycardia might be the only SLE manifestation involving the conduction system, and it usually resolves following treatment with steroids [7]. In this review, we discuss the most commonly occurring cardiac manifestations in patients with SLE and explain if there is any relationship with antibodies present in both adult and neonatal populations.

## Review

Conduction abnormalities in neonates

Neonatal lupus is a consequence of trans-placental migration of maternal immunoglobulin G (IgG) autoantibodies to SSA and/or SSB autoantigens. Autoantibodies identified are of the IgG subtype [6]. This induces serious, even fatal, heart blocks ranging from subclinical first-degree heart blocks to complete heart blocks [7].

Finding conduction defects in neonates without anatomical abnormalities of the heart suggests asymptomatic mothers having anti-SSA/Ro and anti-SSA/La antibodies [8]. Heart blocks in newborns are not related to such diseases in mothers. Congenital cardiac blocks are diagnosable in-utero or in the early neonatal period, and 80-90% of these are due to neonatal lupus [9]. Morbidity and mortality are considerable, at 60% and 30%, respectively, requiring lifelong cardiac pacing in the majority of cases [10]. It is important to detect congenital blocks as early as possible by fetal kinetocardiogram and/or echocardiogram to document atrioventricular (AV) conduction blocks as early as 14-24 weeks [11]. Mortality rates depend on gestational age at birth, with worse prognosis if born before 34 weeks, increasing to 52% vs. 9% if born later. Infants with first- or second-degree blocks at birth can progress to a complete heart block [8]. For patients with lower degrees of heart block, fluorinated steroids, dexamethasone, or betamethasone are the main therapies [12], despite potential adverse reactions to perinatal steroids during neurodevelopment. Steroids are not recommended to treat complete heart block cases and may not be curative [13]. There is a high rate of spontaneous recovery from sinus rhythm in those with transient first-degree congenital heart block [14]. A pacemaker is installed in the majority of the cases, immediately after birth [15]. However, heart failure develops even after pacemaker insertion in 5-11% of the patients [16]. The mechanism for the progression of conduction defects in neonatal lupus is not clear. Endocardial fibroelastosis, ventricular dilatation, and/or ventricular asynchrony can occur and lead to syncope, sudden cardiac death, and heart failure [16,17]. Nevertheless, in most cases, the prognosis is excellent beyond a few months [15]. Table 1 lays out all cardiac conduction defects that are observed in neonatal lupus.

**Table 1:** Cardiac conduction abnormalities observed in neonatal lupus* *[18] AV: atrioventricular

Abnormalities
1^st^ degree AV block
2^nd^ degree AV block
Complete heart block
Atrial and ventricular ectopic beats
Atrial flutter
Ventricular and Junctional tachycardia
Long QT syndrome
Sinus node dysfunction

Conduction abnormalities in adults

1) Autoantibodies

Adults with SLE also can exhibit a variety of conductions. Tachyarrhythmia, bradyarrhythmias, AV nodal blocks (all types), and QT prolongation have been reported and described in the literature [19]. Anti-Ro/SSA antibodies are detected in numerous connective tissue disorders [20]; however, unlike in neonatal lupus, autoantibodies may have a less clear pathological role in adults. Normal people may carry these antibodies in a subclinical form in up to 3% of the general population [21]. There might be no relationship between these antibodies and conduction defects in mothers whose babies were born with neonatal lupus-induced congenital heart blocks [22]. Among patients with SLE having a correlation of anti-Ro antibodies to conduction defects, QT prolongations and non-specific ST-segment elevations are the most common ones [23,24]. QT prolongation is an important focus of attention since it can result in fatal arrhythmias, irrespective of autoimmunity [25]. Studies revealed a mortality association with subtle electrocardiographic changes when SLE patients were followed for 10 [26], 20 [27], and 30 [28] years. Sudden death was the fourth most common cause of death among SLE patients [27]. There is an association between anti-RO/SSA antibodies and QT prolongation as reported in an SLE patient population [29]. Antibodies act on L-type and T-type calcium channels and potassium channels that lead to congenital heart block, and age-related repolarization anomalies in adults are likely [30]. Table 2 depicts studies for and against an association between anti-Ro/SSA antibodies and repolarization anomalies.

Other than QT prolongation, heart blocks are rarely observed in adults with SLE. Some case reports of such patients are listed in Table 2, including a variable AV nodal block in a patient, where a type I block progressed to a type II block [31].

**Table 2:** Studies and case reports of systemic lupus erythematosus-related conduction system anomalies AV: atrioventricular; RNP: ribonucleoprotein

Study/case report	Conduction defect (including repolarization)	Cause (autoantibodies/other)
Cardoso et al. [32]	Prolonged QTc calculated by Bazzet’s formula	Indeterminate
Paradiso et al. [33]	Late excitation potentials	Indeterminate
Guzman et al. [34]	Sinus tachycardia	Indeterminate
Yavuz et al. [35]	Prolonged Qtc	Associated with anti-Ro/SSA antibodies
Pineau et al. [29]	Prolonged Qtc	Associated with anti-Ro/SSA antibodies
Kong et al. [36]	Ventricular ectopy, atrial fibrillation	Associated with myocarditis and pericarditis
Costedoat-C et al. [37]	Prolonged QT	No significant difference in mean QTc duration in association with the presence of antibodies
Gordon et al. [22]	Prolonged QT	No significant difference in mean QTc duration in association with the presence of antibodies
Maier et al. [38] (case report)	Third-degree AV block	Presence of anti-Ro/SSA antibodies in serum
Lim et al. [39] (case report)	Third-degree AV block	Presence of anti-Ro/SSA antibodies in serum
Arce-S et al. [40](case report)	Third-degree AV block	Presence of anti-Ro/SSA antibodies in serum
Mevorach et al. [41](case report)	Third-degree AV block	Presence of anti-Ro/SSA antibodies in serum
Edwards et al. [42]	Third-degree AV block	Presence of anti-Ro/SSA antibodies in serum
Martinez-C et al. [43]	Third-degree AV block	Presence of anti-Ro/SSA antibodies in the serum of one of the two subjects
Bilazarian et al. [44] (case report)	Third-degree AV block	Presence of nuclear RNP antibodies (anti-U1-RNP)

2) Other Causes

SLE can affect many cardiac structures. Myocarditis is an asymptomatic manifestation of lupus, occurring in 8-25% of patients with SLE [45]. Cardiac conduction changes include sinus tachycardia and/or non-specific ST-T wave abnormalities [46,47]. Nevertheless, mortality is higher: 19% vs. 8% in clinical myocarditis vs. subclinical forms. Myocarditis with hemodynamic compromise is a major cause of mortality [48]. A chronic resting sinus tachycardia is associated with augmented lupus activity as depicted by an elevated higher SLE disease activity index [49].

Coronary insufficiency can lead to acute coronary syndromes and sudden death due to arrhythmogenesis [50]. SLE also leads to premature atherosclerosis due to inducing inflammation. Controlling these risk factors might mitigate the atherosclerosis process.

Discussion

SLE causes profound morbidity and mortality. Cardiac conduction defects occur in adults as they do in neonates, but the adult conduction defects are more complex. Repolarization anomalies and subclinical echocardiographic changes are the main manifestations of the disease conduction system [23,24]. It is also clear that autoantibodies are not sensitive to depict conduction anomalies nor vice-versa. It should be noted that the studies described above are low in statistical power. Observing a larger population for a research study is difficult since SLE affects the cardiac conduction system less than other organs; nevertheless, it may affect the myocardium, pericardium, valves, and cause coronary arteries conduction defects. Most patients are prescribed steroids or hydroxychloroquine to suppress the disease before a conduction abnormality presents. A causal relationship to serum markers may or may not be discovered. Cardiac conduction defects occur among patients with SLE and range from sinus tachycardias to prolonged QT-induced ventricular arrhythmias. Larger studies are still needed to reach a conclusive understanding.

## Conclusions

SLE with cardiac manifestations can be seen in 50% of cases. In neonates, various cardiac conduction anomalies are manifested; early detection and installation of a cardiac pacemaker are required in most cases for better prognosis. The most common adult cardiac manifestation of SLE are QT prolongation, non-specific ST-segment elevations, and myocarditis. Our review of the literature has shown the presence of anti-Ro/SSA antibodies and conduction defects.

## References

[1] Petri M, Spence D, Bone LR, Hochberg MC (1992). Coronary artery disease risk factors in the Johns Hopkins Lupus Cohort: prevalence, recognition by patients, and preventive practices. Medicine (Baltimore).

[2] Moder KG, Miller TD, Tazelaar HD (1999). Cardiac involvement in systemic lupus erythematosus. Mayo Clin Proc.

[3] Wallace DJ, Hahn B (2007). Dubois' Lupus Erythematosus. https://books.google.com/books?id=tj9JR3IITy8C&lpg=PR7&ots=nnjaMSAdiM&dq=Dubois'%20lupus%20erythematosus.%202007&lr&pg=PA632#v=onepage&q=Dubois'%20lupus%20erythematosus.%202007&f=false.

[4] Story CM, Mikulska JE, Simister NE (1994). A major histocompatibility complex class I-like Fc receptor cloned from human placenta: possible role in transfer of immunoglobulin G from mother to fetus. J Exp Med.

[5] Guzmán J, Cardiel MH, Arce-Salinas A, Alarcón-Segovia D (1994). The contribution of resting heart rate and routine blood tests to the clinical assessment of disease activity in systemic lupus erythematosus. J Rheumatol.

[6] Capone C, Buyon JP, Friedman DM, Frishman WH (2012). Cardiac manifestations of neonatal lupus: a review of autoantibody-associated congenital heart block and its impact in an adult population. Cardiol Rev.

[7] Nield LE, Silverman ED, Taylor GP (2002). Maternal anti-Ro and anti-La antibody-associated endocardial fibroelastosis. Circulation.

[8] Buyon JP, Hiebert R, Copel J (1998). Autoimmune-associated congenital heart block: demographics, mortality, morbidity and recurrence rates obtained from a national neonatal lupus registry. J Am Coll Cardiol.

[9] Buyon JP, Clancy RM, Friedman DM (2009). Cardiac manifestations of neonatal lupus erythematosus: guidelines to management, integrating clues from the bench and bedside. Nat Clin Pract Rheumatol.

[10] Waltuck J, Buyon JP (1994). Autoantibody-associated congenital heart block: outcome in mothers and children. Ann Intern Med.

[11] Rein AJ, O'Donnell C, Geva T (2002). Use of tissue velocity imaging in the diagnosis of fetal cardiac arrhythmias. Circulation.

[12] Saleeb S, Copel J, Friedman D, Buyon JP (1999). Comparison of treatment with fluorinated glucocorticoids to the natural history of autoantibody-associated congenital heart block: retrospective review of the research registry for neonatal lupus. Arthritis Rheum.

[13] Friedman DM, Kim MY, Copel JA, Llanos C, Davis C, Buyon JP (2009). Prospective evaluation of fetuses with autoimmune-associated congenital heart block followed in the PR Interval and Dexamethasone Evaluation (PRIDE) Study. Am J Cardiol.

[14] Sonesson SE, Salomonsson S, Jacobsson LA, Bremme K, Wahren-Herlenius M (2004). Signs of first-degree heart block occur in one-third of fetuses of pregnant women with anti-SSA/Ro 52-kd antibodies. Arthritis Rheum.

[15] Eliasson H, Sonesson SE, Salomonsson S, Skog A, Wahren-Herlenius M, Gadler F; Swedish Congenital Heart Block Study Group (2015). Outcome in young patients with isolated complete atrioventricular block and permanent pacemaker treatment: A nationwide study of 127 patients. Heart Rhythm.

[16] Udink ten Cate FE, Breur JM, Cohen MI (2001). Dilated cardiomyopathy in isolated congenital complete atrioventricular block: early and long-term risk in children. J Am Coll Cardiol.

[17] Thambo JB, Bordachar P, Garrigue S (2004). Detrimental ventricular remodeling in patients with congenital complete heart block and chronic right ventricular apical pacing. Circulation.

[18] Hornberger LK, Al Rajaa N (2010). Spectrum of cardiac involvement in neonatal lupus. Scand J Immunol.

[19] Bharati S, de la Fuente DJ, Kallen RJ, Freij Y, Lev M (1975). Conduction system in systemic lupus erythematosus with atrioventricular block. Am J Cardiol.

[20] Franceschini F, Cavazzana I (2005). Anti-Ro/SSA and La/SSB antibodies. Autoimmunity.

[21] Hayashi N, Koshiba M, Nishimura K (2008). Prevalence of disease-specific antinuclear antibodies in general population: estimates from annual physical examinations of residents of a small town over a 5-year period. Mod Rheumatol.

[22] Gordon PA, Rosenthal E, Khamashta MA, Hughes GR (2001). Absence of conduction defects in the electrocardiograms [correction of echocardiograms] of mothers with children with congenital complete heart block. J Rheumatol.

[23] Cimaz R, Meroni PL, Brucato A, Fesstovà V, Panzeri P, Goulene K, Stramba-Badiale M (2003). Concomitant disappearance of electrocardiographic abnormalities and of acquired maternal autoantibodies during the first year of life in infants who had QT interval prolongation and anti-SSA/Ro positivity without congenital heart block at birth. Arthritis Rheum.

[24] Bourré-Tessier J, Urowitz MB, Clarke AE (2015). Electrocardiographic findings in systemic lupus erythematosus: data from an international inception cohort. Arthritis Care Res (Hoboken).

[25] Moss AJ (1993). Measurement of the QT interval and the risk associated with QTc interval prolongation: a review. Am J Cardiol.

[26] Godeau P, Guillevin L, Fechner J, Bletry O, Herreman G (1981). Disorders of conduction in lupus erythematosus : frequency and incidence in a group of 112 patients (author's transl). (Article in French). Ann Med Interne (Paris).

[27] Abu-Shakra M, Urowitz MB, Gladman DD, Gough J (1995). Mortality studies in systemic lupus erythematosus. Results from a single center. II. Predictor variables for mortality. J Rheumatol.

[28] Xie SK, Feng SF, Fu H (1998). Long term follow-up of patients with systemic lupus erythematosus. J Dermatol.

[29] Bourré-Tessier J, Clarke AE, Huynh T, Bernatsky S, Joseph L, Belisle P, Pineau CA (2011). Prolonged corrected QT interval in anti-Ro/SSA-positive adults with systemic lupus erythematosus. Arthritis Care Res (Hoboken).

[30] Lazzerini PE, Capecchi PL, Laghi-Pasini F (2010). Anti-Ro/SSA antibodies and cardiac arrhythmias in the adult: facts and hypotheses. Scand J Immunol.

[31] Liautaud S, Khan AJ, Nalamasu SR, Tan IJ, Onwuanyi AE (2005). Variable atrioventricular block in systemic lupus erythematosus. Clin Rheumatol.

[32] Cardoso CR, Sales MA, Papi JA, Salles GF (2005). QT-interval parameters are increased in systemic lupus erythematosus patients. Lupus.

[33] Paradiso M, Gabrielli F, Masala C (2001). Evaluation of myocardial involvement in systemic lupus erythematosus by signal-averaged electrocardiography and echocardiography. Acta Cardiol.

[34] Guzmán J, Cardiel MH, Arce-Salinas A, Alarcón-Segovia D (1994). The contribution of resting heart rate and routine blood tests to the clinical assessment of disease activity in systemic lupus erythematosus. J Rheumatol.

[35] Yavuz B, Atalar E, Karadag O (2007). QT dispersion increases in patients with systemic lupus erythematosus. Clin Rheumatol.

[36] Kong TQ, Kellum RE, Haserick JR (1962). Clinical diagnosis of cardiac involvement in systemic lupus erythematosus. A correlation of clinical and autopsy findings in thirty patients. Circulation.

[37] Costedoat-Chalumeau N, Amoura Z, Hulot JS, Ghillani P, Lechat P, Funck-Brentano C, Piette JC (2005). Corrected QT interval in anti-SSA-positive adults with connective tissue disease: comment on the article by Lazzerini et al. Arthritis Rheum.

[38] Maier WP, Ramirez HE, Miller SB (1987). Complete heart block as the initial manifestation of systemic lupus erythematosus. Arch Intern Med.

[39] Lim LT, Joshua F (2005). Resolution of complete heart block after prednisolone in a patient with systemic lupus erythematosus. Lupus.

[40] Arce-Salinas CA, Carmona-Escamilla MA, Rodríguez-García F (2009). Complete atrioventricular block as initial manifestation of systemic lupus erythematosus. Clin Exp Rheumatol.

[41] Mevorach D, Raz E, Shalev O, Steiner I, Ben-Chetrit E (1993). Complete heart block and seizures in an adult with systemic lupus erythematosus. A possible pathophysiologic role for anti-SS-A/Ro and anti-SS-B/La autoantibodies. Arthritis Rheum.

[42] Edwards CS, Mootoo R, Bhanji A (2004). High grade heart block in association with SLE. Ann Rheum Dis.

[43] Martinez-Costa X, Ordi J, Barberá J, Selva A, Bosch J, Vilardell M (1991). High grade atrioventricular heart block in 2 adults with systemic lupus erythematosus. J Rheumatol.

[44] Bilazarian SD, Taylor AJ, Brezinski D, Hochberg MC, Guarnieri T, Provost TT (1989). High-grade atrioventricular heart block in an adult with systemic lupus erythematosus: the association of nuclear RNP (U1 RNP) antibodies, a case report, and review of the literature. Arthritis Rheum.

[45] Tincani A, Rebaioli CB, Taglietti M, Shoenfeld Y (2006). Heart involvement in systemic lupus erythematosus, anti-phospholipid syndrome and neonatal lupus. Rheumatology (Oxford).

[46] Perel-Winkler A, Bokhari S, Perez-Recio T, Zartoshti A, Askanase A, Geraldino-Pardilla L (2018). Myocarditis in systemic lupus erythematosus diagnosed by 18F-fluorodeoxyglucose positron emission tomography. Lupus Sci Med.

[47] Zhang L, Zhu YL, Li MT (2015). Myocarditis: a case-control study from China. Chin Med J (Engl).

[48] Apte M, McGwin G Jr, Vilá LM, Kaslow RA, Alarcón GS, Reveille JD; LUMINA Study Group (2008). Associated factors and impact of myocarditis in patients with SLE from LUMINA, a multiethnic US cohort (LV). Rheumatology (Oxford).

[49] Spodick DH, Raju P, Bishop RL, Rifkin RD (1992). Operational definition of normal sinus heart rate. Am J Cardiol.

[50] Ghuran AV, Camm AJ (2001). Ischaemic heart disease presenting as arrhythmias. Br Med Bull.

